# Novel Opportunities for Cathepsin S Inhibitors in Cancer Immunotherapy by Nanocarrier-Mediated Delivery

**DOI:** 10.3390/cells9092021

**Published:** 2020-09-02

**Authors:** Natalie Fuchs, Mergim Meta, Detlef Schuppan, Lutz Nuhn, Tanja Schirmeister

**Affiliations:** 1 Institute of Pharmaceutical and Biomedical Sciences, Johannes Gutenberg University of Mainz, Staudingerweg 5, D, 55128 Mainz, Germany; nafuchs@uni-mainz.de (N.F.); meta@uni-mainz.de (M.M.); 2 Institute of Translational Immunology and Research Center for Immunotherapy (FZI), University Medical Center of the Johannes Gutenberg-University Mainz, Obere Zahlbacher Str. 63, 55131 Mainz, Germany; 3 Division of Gastroenterology, Beth Israel Deaconess Medical Center, Harvard Medical School, 330 Brookline Avenue, Boston, MA 02215, USA; 4 Max Planck Institute for Polymer Research Ackermannweg 10, 55128 Mainz, Germany

**Keywords:** cysteine protease, cysteine cathepsin, nanoparticle, tumor microenvironment, immune suppression, therapy, targeting, tumor associated macrophage, dendritic cell, T cell, antigen presentation, antigen presenting cell, extracellular matrix (ECM), polarization, M2 macrophage, tumor-associated macrophage (TAM)

## Abstract

Cathepsin S (CatS) is a secreted cysteine protease that cleaves certain extracellular matrix proteins, regulates antigen presentation in antigen-presenting cells (APC), and promotes M2-type macrophage and dendritic cell polarization. CatS is overexpressed in many solid cancers, and overall, it appears to promote an immune-suppressive and tumor-promoting microenvironment. While most data suggest that CatS inhibition or knockdown promotes anti-cancer immunity, cell-specific inhibition, especially in myeloid cells, appears to be important for therapeutic efficacy. This makes the design of CatS selective inhibitors and their targeting to tumor-associated M2-type macrophages (TAM) and DC an attractive therapeutic strategy compared to the use of non-selective immunosuppressive compounds or untargeted approaches. The selective inhibition of CatS can be achieved through optimized small molecule inhibitors that show good pharmacokinetic profiles and are orally bioavailable. The targeting of these inhibitors to TAM is now more feasible using nanocarriers that are functionalized for a directed delivery. This review discusses the role of CatS in the immunological tumor microenvironment and upcoming possibilities for a nanocarrier-mediated delivery of potent and selective CatS inhibitors to TAM and related APC to promote anti-tumor immunity.

## 1. Introduction

Intra- and extracellular protein degradation is central to the maintenance of homeostasis in health and disease and therefore needs to be tightly controlled. Chronic diseases, especially autoimmunity, organ fibrosis, and cancer are usually characterized by dysregulated proteolysis that contributes to disease progression. The lysosomal proteases are involved in protein catabolism and some of them, prominently several cysteine cathepsins, are overexpressed in tumors [1,2,3]. This makes them attractive targets in the development of new anti-cancer drugs. 

**Figure 1:**
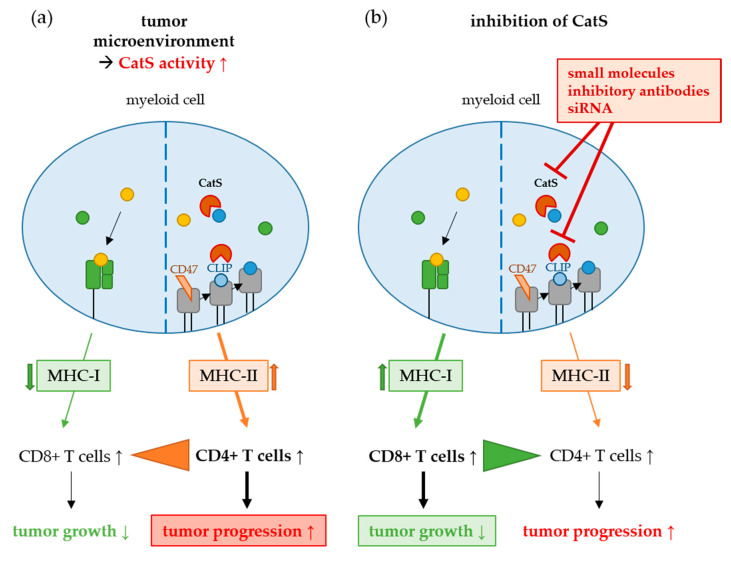
Role of cathepsin S (CatS) in the immune-suppressive milieu of the tumor microenvironment (TME). (**a**) CatS is overexpressed in many tumors and favors the MHC-II pathway, leading to an increase of CD4+ T cells instead of cytotoxic CD8+ T cells. (**b**) The inhibition of CatS can enhance the anti-tumor immune response by promoting cytotoxic CD8+ T cells instead of CD4+ T cells [1,3].

Cysteine cathepsins comprise a family of 11 proteases, of which 5 have been repeatedly implicated in the progression of solid cancers (cathepsins B, H, K, L, and S) [4]. Among these, cathepsin S (CatS) has emerged as an attractive potential target whose inhibition promises to address the immune-suppressive milieu of the tumor microenvironment (TME) [1,3,4] due to its role in the polarization of antigen-presenting cells (APC) from an M1 toward a tumor-favoring M2-phenotype. M2-type APC support myeloid-derived suppressor cells (MDSC) and tumor-associated macrophages (TAM), favoring the expansion of tolerogenic regulatory T cells (Treg) cells instead of cytotoxic CD8+ T cells, which leads to an immune suppression in favor of tumor cells (Figure 1) [5]. 

In this review, the mechanisms of tumor promotion by cysteine cathepsins, with a focus on CatS and its role in the TME, will be illustrated. Several inhibitors of CatS and strategies for efficient delivery into the TME, mediated by nanoparticles, will be presented.

## 2. Cysteine Cathepsins in Tumor Progression

Cysteine cathepsins (Cat) are lysosomal proteases, usually with an activity optimum at an acidic pH, that play an important role in intracellular protein catabolism. The cysteine cathepsins B, H, K, L, and S are also excreted, partly tethered to the cell surface, where they also degrade certain extracellular matrix (ECM) proteins. For CatS, these are several collagens, elastin, laminin-5, but also cell surface receptors, such as protease-activated receptor-2, and cell adhesion molecules such as junctional adhesion molecule B (JAM-B) and E-cadherin, which is an activity that facilitates tissue remodeling, cancer cell growth, and spreading ([4] Table 1). The expression and activity of these cathepsins is generally upregulated in (chronic) inflammation and in cancers. Consequently, they are overexpressed in tumors, prominently for CatB and CatS, including in follicular lymphoma, gastric, colon, brain, breast, and pancreatic cancer [1,3,6]. Overall, most but not all of these cathepsin-mediated mechanisms result in enhanced ECM turnover and angiogenesis, clearing the way for tumor expansion, securing the cancers’ nutrient supply, and, most notably, in suppressing the T-cell induced anti-cancer immune response that is located in the TME. Here, cancer cells have developed mechanisms to escape the surveillance of the immune system, both by limiting their (tumor) antigen presentation which makes them “invisible” to the immune system and by their ability to actively condition their TME by the secretion of factors that switch the non-tumor antigen-presenting cells (APC), mainly myeloid cells and partly B cells, from an M1-type to an M2-type polarization. M1-type APC activate tumor destroying CD8+ T cells, while M2-type APC induce tumor tolerogenic regulatory T cells (Treg) and inactivate tumor destroying cytotoxic CD8+ T cells [3,7,8]. Here, the proteolytic activity of mainly CatB and CatS, despite their complex regulation and diverse activities, has an overall disastrous effect on anti-tumor immune responses by polarizing the myeloid APC in the TME from an M1-type toward an M2-phenotype, favoring the expansion and suppressive function of myeloid-derived suppressor cells (MDSC) and the related tumor-associated macrophages (TAM), resulting in the inactivation and depletion of cytotoxic CD8+ T cells and the expansion of Treg [5]. CatS regulates antigen processing and presentation, enhancing major histocompatibility class II (MHCII) expression and antigen loading on myeloid endothelial and epithelial cells, including cancer epithelia. However, in contrast to antigen presentation via MHC class I, prominently via (cancer) epithelial cells, that activate tumor cytotoxic CD8+ T cells, MHCII presentation in the TME usually activates CD4+ Treg that actively suppress cancer immunity [9,10,11,12]. Furthermore, a mouse model suggests that the CatS inhibition of Treg cells may reduce the overall T-cell immunity under normal conditions, but it enhances the CD8+ T-cell immunity in the presence of cancer cells [13]. Taken together, all these effects of CatS on cancer growth make the inhibition of CatS an attractive strategy to limit tumor expansion and increase anti-tumor immunogenicity [3,9,10,11].

### Cathepsin S

**Figure 2:**
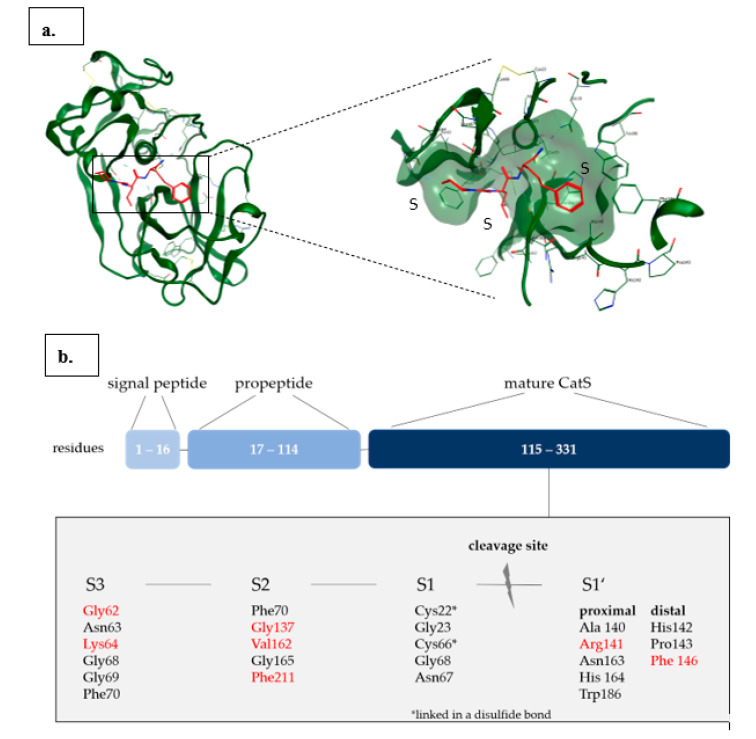
(**a**) Left: Cartoon model of the crystal structure of CatS in green (PDB 1MS6) in complex with a covalent-reversible nitrile-based inhibitor in red bound covalently to the catalytic Cys25 of CatS. Right: zoom into the active site of CatS with hydrogen bonds depicted in dashed lines. The subsites S1, S2, and S3 are highlighted. (**b**) Domain structure of CatS. The residues that define the S1’–S3 pockets are listed in the light gray box; residues that are the determinants of CatS binding specificity among papain-like cysteine endopeptidases are marked in red [32]. Figure 1a was prepared with MOE 2019.0102.

Cathepsin S (CatS) is a papain-like two-domain protein that is synthesized in vivo as an inactive precursor. Its propeptide is essential for the activation of the enzyme and proper folding [29,30]. The mature enzyme contains 217 residues with a catalytic Cys25 located in the active site and several residues in the S1’–S3 pockets that determine the binding specificity for CatS among other papain-like cysteine endopeptidases (Figure 2) [31,32].

Although the different cathepsin subtypes have a high sequence homology, CatS differs from other cysteine cathepsins by its stability at neutral pH and its limited tissue distribution with high levels found in spleen and lung macrophages of healthy organisms [33,34]. CatS degrades the invariant chain that occupies the MHCII binding pocket and promotes (tumor) antigen processing and their presentation to T cells via MHCII on APC [9]. However, as discussed above, this activates immune-suppressive CD4+ Treg instead of tumor-destructive CD8+ T cells.

Recently, the effect of CatS inhibition in several experimental cancers has been demonstrated. For instance, Da Costa et al. explored the inhibition of CatS in gastric cancer, where it is expressed not only in the lysosomes but also secreted into the ECM. CatS silencing via small interfering RNA (siRNA) led to a reduction of tumor volume and invasion accompanied by increased apoptosis and attenuated angiogenesis [35,36]. Sevenich et al. claimed that CatS specifically mediates the blood–brain barrier transmigration of breast cancer cells through proteolytic processing of the junctional adhesion molecule B (JAM-B) and therefore, it plays an important role in brain metastasis [37]. Additionally, Yang et al. showed that the increased expression of CatS correlates with the aggressiveness of human colon cancer due to its promotion of the M2-type macrophage polarization (TAM), which is favored by CatS-induced autophagy [38]. Moreover, preclinical studies by Burden et al. illustrate the impact of inhibitory antibodies against CatS, resulting in an increased efficacy of chemotherapeutic treatments, leading to a significant reduction of tumor growth [39,40]. Notably, CatS appears to play an important role in liver cancer that is otherwise resistant to most conventional tumor therapies [41,42,43].

Bararia et al. reported an overexpression of CatS and its hyperactive mutant Y132D in follicular lymphoma. The Y132D mutation results in a higher autocatalytic conversion from the inactive proform to active CatS. In a CatS Y132D transgenic mouse model of follicular lymphoma, they observed increased cancer growth versus wild-type controls and an increase of the tumor-suppressive CD4+ Treg over cytotoxic CD8+ T cells infiltrating the tumor [1]. In confirmation, Dheilly et al. showed that in patients with non-Hodgkin lymphoma, the same activating CatS Y132D mutation in malignant B cells is promoted, and its inhibition abrogated lymphoma growth via enhancing the cytotoxic CD8+ T-cell response and attenuating the expansion of CD4+ Treg [3]. Hence, the directed inhibition of CatS with small molecule inhibitors could enhance the anti-tumor immune response in cancer, especially when targeting the TME and the relevant APC, i.e., primarily myeloid TAM and MDSC, and—depending on the tumor type—also B cells.

## 3. Cathepsin S Inhibitors

The search for selective CatS inhibitors dates back to the early 2000s and continues until today. Some early reviews [44,45,46,47] cover the inhibition of cysteine proteases in general, while the first specific compilation of selective CatS inhibitors appeared in 2004 [48]. CatS has a high sequence homology with other Cathepsins (K, L, B), with some distinct differences in the S2 and S3 pockets which can be addressed for selective CatS inhibition [32]. The S2 pocket of CatS contains Phe70, Gly137, Val162, Gly 165, and a flexible Phe211 located at the bottom of the pocket, which can lead to an open conformation making space between itself and Phe70 and providing a possibility for π-stacking interactions with either or both phenylalanine residues [48]. In cathepsin K (CatK), Phe211 is replaced by Leu, making the subsite tighter and more shallow. The two Gly residues in CatS are both Ala in Cat K and serve as ‘gatekeepers’. The missing methyl groups of the two Gly amino acids instead of Ala in CatS enable the S2 pockets to be opened in width and in depth [32,49,50]. These differences make the search for selective CatS inhibitors attractive, since the S2 pocket in CatS can be addressed by significantly lager substituents compared to other cathepsins, e.g., CatK [49].

**Figure 3:**
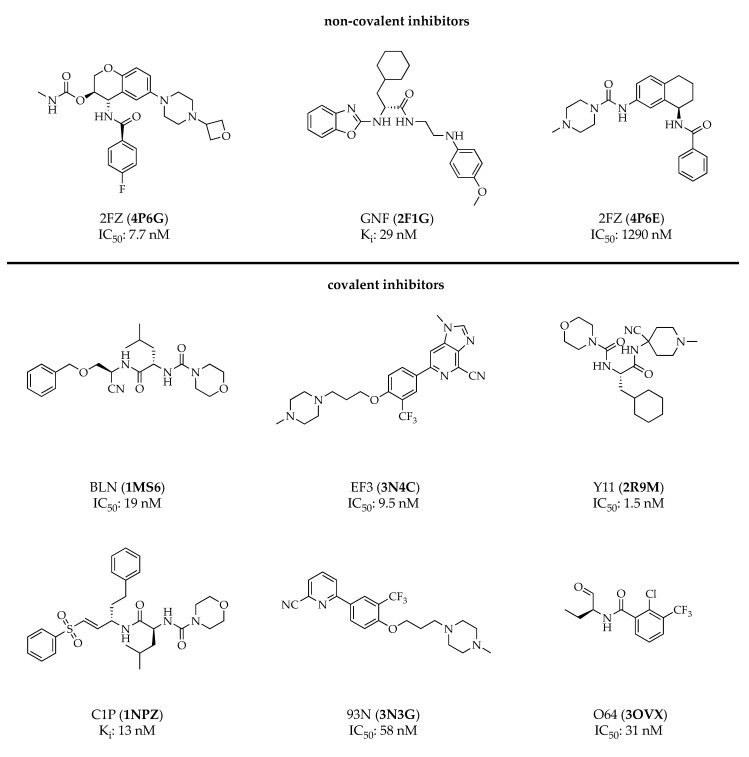
Overview of different non-covalent and covalent CatS-selective inhibitors from crystal structures with compound ID and PDB (protein database) code of the corresponding crystal structure (in parenthesis). The enzymatic data are given as either IC_50_ or K_i_ values [53,54,55,56,57,58].

To date, there are over 1800 entries in the public access ZINC (ZINC is not commercial) database regarding CatS inhibitors [51], most of them being non-selective and inhibiting other cathepsins as well. The most selective and potent compounds developed for CatS inhibition comprise covalent as well as non-covalent inhibitors [48,50,52]. Since the first investigated inhibitors for CatS, e.g., the pan-cathepsin inhibitor LHVS (leucine homophenylalanine vinyl sulfone), contained an electrophilic warhead, which bound to the catalytical cysteine residue in the active site of CatS in a covalent irreversible mechanism, a wide variety of covalent reversible and interestingly also non-covalent inhibitors have been developed [48]. This has opened up new possibilities in CatS inhibitor design. Most of the covalent inhibitors contain a nitrile or an aldehyde as an electrophilic warhead, and the recognition sequence harbors either peptidic or non-peptidic residues. Both covalent and non-covalent inhibitors were successfully co-crystallized with CatS (Figure 3) and many of those crystal structures are published in the protein database (PDB) [53,54,55,56,57,58]. Since the latest reviews of CatS inhibitors covered the state of research in CatS inhibitor design for the time between early 2000 to 2010, this paragraph focuses on the progress made in the last decade [48,50,52].

**Figure 4:**
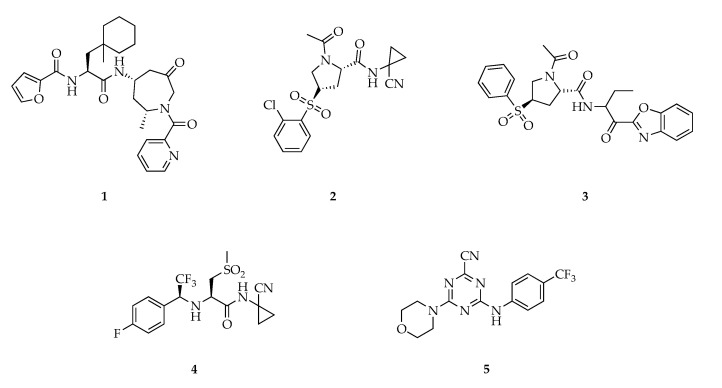
Structures of five relevant CatS inhibitors discovered through SAR (structure–activity relation) studies of the last decade.

One effort for optimizing the selectivity toward CatS via P2 substituents was done by Kerns et al. reporting azepanone-based inhibitors (Figure 4) with 175-fold and 286-fold selectivity for CatS versus CatK and CatL respectively [59]. The crucial modification in P2 with the introduction of a 1-methyl-cyclohexyl alanine lead to compound 1 with an overall balanced potency and selectivity profile. Another approach has been taken by Hilpert et al. through structure-based drug design starting from a weakly active dual CatS/CatK inhibitor discovering a series of CatS-selective inhibitors that contain different cyclic central scaffolds [57]. Out of the different tested scaffolds, a proline derivative was identified as the most promising with IC_50_ values in the low nM range in enzymatic as well as cell-based assays and a good ligand-binding efficiency (LE) of 0.47 (Figure 4) [57]. Another set of proline-derived compounds was synthesized and evaluated as CatS inhibitors by Kim et al., with compound 3 (Figure 3) showing promising in vitro/in vivo pharmacological activities [60]. A known bioavailable CatS nitrile inhibitor previously developed by Merck Frosst [49] was re-evaluated, and its selective targeting of CatS over CatK/L/V and B was confirmed by an in vitro enzymatic assay. In vivo experiments revealed that compound 4 could significantly reduce tumor volume in murine MC38 syngeneic and MCF7 xenograft models. Immunohistochemical analysis of MCF7 tumors revealed that CatS inhibitor treatment with compound 4 significantly reduced proliferation and increased apoptosis [61].

Thus, this inhibitor may hold therapeutic potential and could be used to further analyze the role of CatS in tumor development. One additional series of reversible CatS inhibitors consisting of 2,4,6-trisubstituted 1,3,5-triazines was synthesized and biologically evaluated by Tber et al., with compound 5 being the most potent and selective inhibitor among the set, with an IC_50_ of 3 nM and a K_i_ of 2 nM, respectively [62].

Overall, there has been progress in the field of CatS inhibitor research resulting in several potent and selective compounds that have been developed in the last decade and that are suitable for the use in further investigations focusing on the role that CatS plays in cancer and also other pathologies.

## 4. Nanocarrier-Mediated Delivery of Cathepsin Inhibitors

Despite pharmacodynamic target optimization, physicochemical parameters may sometimes hamper the in vivo translatability of small molecular drugs, including some of the novel CatS inhibitors discussed above. For instance, poor solubility and reduced bioavailability might already prevent them from entering in vivo testings. Here, nano-sized carrier systems can provide unique opportunities to alter the drugs’ pharmacokinetic profile and enhance their delivery to targeted sites of action. By preventing premature release from the carrier or unwanted early metabolic degradation, nano-sized carrier systems can support the overall drug performance in vivo and at the same time reduce unwanted side effects, which can further enable higher dosing, thus increasing the therapeutic window [63].

Through the last four decades, nanocarriers have intensely been investigated for the delivery of classical anti-cancer chemotherapeutics [64,65]. This has so far mostly been stimulated by blood-circulating nanocarriers that can passively accumulate in highly vascularized tumors through the enhanced permeability and retention (EPR) effect [66,67,68]. In parallel, several studies demonstrated that nano-sized drug carriers also accumulate in lymphoid organs, such as the spleen, but also in the liver and lungs, as major first pass organs, to be rapidly taken up and processed by immune cells, in analogy to classical pathogens of similar sizes, i.e., “the immune system likes nanotechnology” [69,70]. These more recent insights have increased the interest in nanocarriers to assist in cancer immunotherapy [71,72,73]. Here, nanocarrier-mediated delivery of CatS inhibitors to focus their delivery to certain immune cells in the tumor microenvironment, especially to TAM and MDSC, is an attractive approach.

Key criteria for precise delivery to specific immune cell populations include the carriers’ chemical composition, which should guarantee biocompatibility, biostability, and biointegrity under physiological conditions. Moreover, toward clinical translatability, further issues including biodegradability and clearance from the body must be taken into account as well. Consequently, these characteristics needed for in vivo application exclude several nanocarriers that have been designed throughout the last decades and claimed useful for drug delivery purposes [74]. So far, only a few formulations passed phase 1–3 clinical trials and led to successful products on the market. Most prolific amongst them are lipid formulation e.g., liposomal doxorubicine (Doxil) for solid tumors (e.g., breast or ovarian cancer), or the lately approved lipid-encapsulated siRNA formulation Patisiran [75]. Additionally, such formulations can be combined with polymer-based strategies, e.g., PEGylation (poly(ethylene glycol) decoration) of liposomes or therapeutic proteins [76,77], which enable tissue mobility and prolonged blood circulation.

Combining biocompatible hydrophilic polymers such as PEG and hydrophobic, self-assembling block copolymers affords lipid-analogous polymer micelles with superior particle stabilities and drug encapsulation capabilities [78]. As an example, South Korea approved a paclitaxel formulation using PEG-block-poly-lactic-co-glycolic acid (PLGA) block copolymers (Genexol) [79]. Such systems support the solubility of poorly water-soluble drugs by physiochemically entrapping them into the hydrophobic domains of resulting nano-sized block copolymer self-assemblies.

PLGA has been approved by the FDA as synthetic hydrophobic copolymer and therefore, it is frequently used for encapsulating therapeutically active molecules [80]. Consequently, similar attempts have been made lately to encapsulate also cathepsin inhibitors, as recently summarized by Prunk et al. [14] and Cogo et al. [81]. For instance, Kos et al. have encapsulated the endogenous pan-cathepsin inhibitor cystatin, a 13.3 kDa protein, into PLGA microspheres and nanospheres via a double emulsion process [82,83]. They also studied a biological polymer, chitosan, for cystatin encapsulation via a gelation process including polyphosphates [84]. Similarly, the small molecule hydroxyquinoline derivative nitroxoline, which exhibits CatB inhibitory activity, has more recently been encapsulated into nanoparticles via gel-forming chitosan/chondroitin sulfate mixtures that further allowed co-encapsulation of the anti-cancer agent 5-fluoruracil. [85] Other small molecule-based cathepsin inhibitors could be co-formulated into polymeric nanoparticles, too. For instance, the group of Schindeler locally delivered the CatK inhibitor L006235 encapsulated into PLGA nanospheres together with recombinant human bone morphogenetic protein-2 (rhBMP-2) [86], while the group of Prud’homme studied co-formulations of the CatK inhibitor odanacatib with the antifungal drug itraconazole, which are both encapsulated into amphiphilic PEG-block-poly(styrene) nanocarriers [87].

Cathepsin inhibitor delivery has also been studied in liposomal formulations. In analogy to the natural pan-cathepsin inhibitor cystatin, Mikhaylov et al. loaded the small molecule broad spectrum inhibitor JPM-565 into PEGylated liposomes together with ferrimagnetic iron oxide nanoparticles yielding “ferri-liposomes”, which allowed tumor-directed MRI-based imaging, and resulted in local cathepsin inhibition and finally reduced mammary tumor growth [88]. Alternatively, in another study, Mikhaylov et al. covalently attached the small molecule CatB inhibitor NS-629 to a DSPE-PEG (2000) lipid and thus anchored it into the bilayer of resulting liposomes [89]. More lately, the same group also applied this approach for the conjugation of the natural CatS and CatL inhibitor stefin A onto the liposome surface, too [90].

Tabish et al. recently summarized further CatL inhibitors and proposed their delivery by nanocarriers to enhance anti-tumor and antimetastatic activities [91]. As a recent example, Junior et al. reported the encapsulation of the CatL inhibitors Neq0551, Neq0554, and Neq0568 into protein-based hollow ferritin nanocages and demonstrated improved drug delivery into cancer cells in vitro [92].

Taken together, such physicochemical encapsulation methods seem to be straightforward regarding fabrication processes; however, several important challenges, including reproducible loading capacities, avoiding premature drug release and controlled drug release at the target site need to be shown. Especially, a chemically better defined and carefully designed approach to covalently link small molecule drugs to the carrier may be promising. Here, the group of Kopeček synthesized well-defined conjugates of the CatK inhibitor 1,5-bis (N-benzyloxy- carbonylleucyl) carbohydrazide with the water-soluble hydrophilic polymers PEG and poly(2-hydroxypropyl methacrylamide) (HPMA) [93,94]. Yet, ligating water-insoluble small molecules to hydrophilic polymers can stimulate less controlled inter- and intramolecular self-assemblies that affect the nanocarriers’ integrity, especially under biologically relevant conditions, e.g., by a less predictable adsorption of serum proteins [95,96].

**Figure 5:**
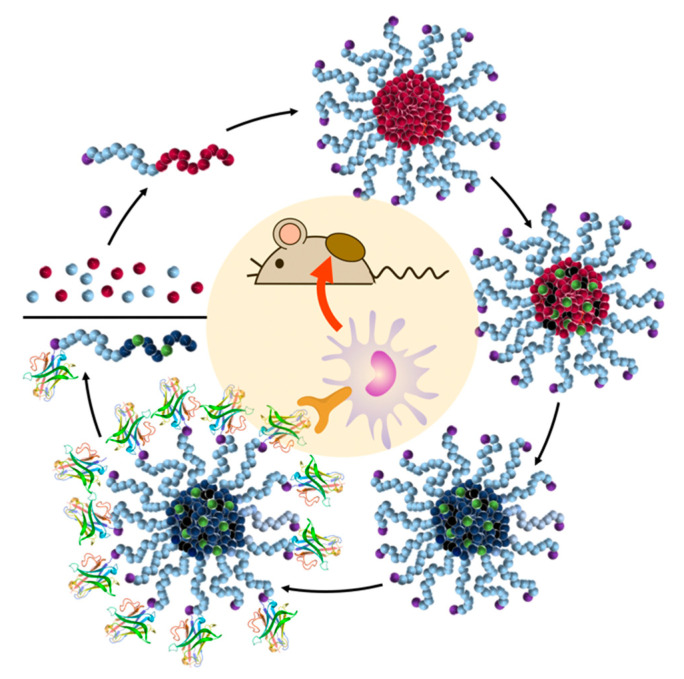
Concept of delivering small molecular drugs such as CatS inhibitors (depicted in green) via covalent attachment to reversibly core-crosslinked and immune cell-targeting nanogels for cancer immunotherapy. Reproduced with permission [99]. Copyright 2020, Elsevier B.V.

To address these challenges, block copolymer micelles have been demonstrated to provide higher drug-loading stability and carrier integrity when interchain cross-links are introduced, especially into the hydrophobic core of the drug nanocarrier [97]. We have recently been following this strategy [98] and optimized the nanocarriers for immunotherapeutic delivery (Figure 5) [99]. Via covalent drug conjugation and hydrophilic core-crosslinking of self-assembled reactive ester block copolymers, drug-loaded nanogels can be obtained with pH- or reductive-responsive degradation profiles [100,101]. Interestingly, these carriers nicely facilitated the antiviral and antitumor immune responses of highly potent small molecular Toll-like receptor 7/8 agonists [102,103,104]. Moreover, surface chemistry modification can also be applied to further promote an active delivery of these carriers to certain target immune cell subpopulations [105].

To that respect, the targeted delivery of cathepsin inhibitors has also been followed by *Kos* et al. by adsorbing anti-cytokeratin antibodies onto their cystatin-loaded PLGA nanosphere systems to enhance delivery to breast cancer cells in vitro [106,107]. However, even more attractive for the novel CatS inhibitors would be targeting their delivery to more relevant immune cells of the TME, i.e., immune-suppressive TAM and MDSC [108,109]. Targeting these cell populations could so far be demonstrated for surface-modified reactive-ester based nanogels, either by using polymers with α-mannosyl end groups binding to the overexpressed TAM mannose (MMR, CD206) receptor in vitro [110] or by targeting this receptor through nanogel-conjugated MMR/CD206-binding nanobodies in vivo [105]. The latter is currently considered to be more effective in terms of cheap recombinant production and precise chemical modification compared to full antibodies or antibody–drug conjugates. More recently, also peptide-based targeting strategies [111,112] or multivalent mannose derivatives [113] have been exploited to guide nanoparticle delivery toward TAM. Interestingly, mannose targeting has also been directly applied to small molecule cathepsin inhibitors by generating glycoconjugates of monomannose, trimannose, and heptamannose with the pan-cathepsin inhibitor DCG-04 [114].

Therefore, targeted delivery strategies of cathepsin inhibitors to immune-suppressive myeloid cells, especially for CatS, create a highly promising approach to a novel anti-cancer treatment. The nanocarrier-mediated delivery of CatS inhibitors has so far not been reported. Consequently, based on the novel, highly specific CatS inhibitors and substantial advances in the development of suitable nanocarriers that show promising performance not only in vitro but also in vivo, we expect that combining both strategies has become an exciting research area for developing novel therapeutics to sustainably suppress cancer growth and progression.

## 5. Conclusion and Outlook

The inhibition of tumor-associated cathepsins, especially cathepsin S (CatS), has emerged as a novel promising strategy in cancer immunotherapy. CatS is prominently expressed in tumor associated M2-type macrophages (TAM), dendritic cells (DC), and myeloid-derived suppressor cells (MDSC) of the TME. Its inhibition downregulates MHC class II expression on TAM and DC, and repolarizes TAM, DC, and MDSC toward the M1 phenotype that promotes the proliferation and activity of CD8+ tumor-destroying cytotoxic T cells, and that abrogates CD4+ immune-suppressive regulatory T cells. A secondary effect is the suppression of tumor angiogenesis. Therefore, further research on CatS selective inhibitors and their targeting to M2-type TAM, DC, and MDSC is warranted. We propose that selective CatS inhibition by specific small molecule inhibitors or siRNA can be targeted to TAM, DC, and MDSC via precisely modified nanocarriers equipped for the directed delivery and functional release of the inhibitors. Such an approach provides novel opportunities for effective adjunctive therapies to promote anti-tumor immunity.

## Figures and Tables

**Table 1:** Cysteine cathepsins and their prominent roles in tumor progression.

	CatB	CatK	CatL	CatS	CatX
Physiological occurrence [14]	ubiquitous	ubiquitous; predominantly in bone tissue	ubiquitous,	ubiquitous, more prominent in M2-type macrophages than T cells, EC, epithelia	predominantly in immune cells [15]
Modified/additional occurrence in tumor tissue [14]	cytoplasm [16,17], plasma membrane, secreted in ECM	-	nucleus, secreted in ECM [18]	TAM > MDSC > angiogenic EC, TM epithelia [5,19]	-
Mechanisms of tumor progression	angiogenesis [20]	angiogenesis, bone metastasis [21]	metastasis, cell proliferation [18]	ECM turnover, angiogenesis [22], suppression of anti-tumor immune responses ↓ [3,5]	additive effects of CatB + CatX [15]
Functions in immune response [5]	CD8+ cell apoptosis [23], MDSC promotion [24]	secretion of IL-6 [19], enhanced expression of COX2 + CatB (via CCL2) [25]	antigen presentation + processing [12]	antigen presentation + processing [26], M2-type macrophage polarization toward TAM [27]	enhanced migration of T lymphocytes [28]
Influence on anti-tumor immune response	↓	↓↑	↑	↓	↑

EC = endothelial cell; ECM = extracellular matrix; MDSC = myeloid-derived suppressor cells; IL-6 = interleukin 6; COX2 = cyclooxygenase 2, CCL2 = CC-chemokine ligand-2; TAM = tumor-associated macrophage.

## Abbreviations

APC	antigen-presenting cell;
Cat	cathepsin;
CatB	cathepsin B;
CatK	cathepsin K;
CatL	cathepsin L;
CatS	cathepsin S;
CCL2	CC-chemokine ligand-2;
CD206	cluster of differentiation 206;
COX2	cyclooxygenase 2;
DC	dendritic cell;
EC	endothelial cell;
ECM	extracellular matrix;
EPR	enhanced permeability and retention;
FDA	Food and Drug Administration;
HPMA	poly(2-hydroxypropyl methacrylamide);
IL-6	interleukin 6;
JAM-B	junctional adhesion molecule B;
LE	ligand efficiency;
LHVS	leucine homophenylalanine vinyl sulfone;
MC38	murine colon adenocarcinoma;
MCF7	Michigan Cancer Foundation–7;
MDSC	myeloid-derived suppressor cell;
MHC	major histocompatibility complex;
MMR	mannose receptor;
MRI	magnetic resonance imaging;
PDB	protein database;
PEG	polyethylene glycol;
PLGA	PEG-block-poly-lactic-co-glycolic acid;
rhBMP-2	recombinant human bone morphogenetic protein-2;
SAR	structure–activity relationship;
siRNA	small interfering RNA;
TAM	tumor-associated macrophages;
TME	tumor microenvironment;
Treg	regulatory T cell;
ZINC	ZINC is not commercial;

## References

[1] Bararia D., Hildebrand J.A., Stolz S., Haebe S., Alig S., Trevisani C.P., Osorio-Barrios F., Bartoschek M.D., Mentz M., Pastore A. (2020). Cathepsin S Alterations Induce a Tumor-Promoting Immune Microenvironment in Follicular Lymphoma. Cell Rep..

[2] Da Costa A.C., Santa-Cruz F., Mattos L.A.R., Aquino M.A.R., Martins C.R., Ferraz Álvaro A.B., Figueiredo J.L. (2019). Cathepsin S as a target in gastric cancer (Review). Mol. Clin. Oncol..

[3] Dheilly E., Battistello E., Katanayeva N., Sungalee S., Michaux J., Duns G., Wehrle S., Sordet-Dessimoz J., Mina M., Racle J. (2020). Cathepsin S Regulates Antigen Processing and T Cell Activity in Non-Hodgkin Lymphoma. Cancer Cell.

[4] Wilkinson R., Williams R., Scott C.J., Burden R.E., Williams R. (2015). Cathepsin S: Therapeutic, diagnostic, and prognostic potential. Biol. Chem..

[5] Jakoš T., Pišlar A., Jewett A., Kos J. (2019). Cysteine Cathepsins in Tumor-Associated Immune Cells. Front. Immunol..

[6] McDowell S.H., Gallaher S.A., Burden R.E., Scott C.J. (2020). Leading the invasion: The role of Cathepsin S in the tumour microenvironment. Biochim. Et Biophys. Acta (BBA) Bioenerg..

[7] Farhood B., Najafi M., Mortezaee K. (2018). CD8+ cytotoxic T lymphocytes in cancer immunotherapy: A review. J. Cell. Physiol..

[8] Quaranta V., Schmid M.C. (2019). Macrophage-Mediated Subversion of Anti-Tumour Immunity. Cells.

[9] Riese R.J., Wolf P.R., Brömme D., Natkin L.R., Villadangos J.A., Ploegh H.L., Chapman H.A. (1996). Essential Role for Cathepsin S in MHC Class II–Associated Invariant Chain Processing and Peptide Loading. Immunity.

[10] Riese R.J., Mitchell R.N., Villadangos J.A., Shi G.P., Palmer J.T., Karp E.R., De Sanctis G.T., Ploegh H.L., Chapman H.A. (1998). Cathepsin S activity regulates antigen presentation and immunity. J. Clin. Investig..

[11] Shi G.-P., Villadangos J.A., Dranoff G., Small C., Gu L., Haley K.J., Riese R., Ploegh H.L., Chapman H.A. (1999). Cathepsin S Required for Normal MHC Class II Peptide Loading and Germinal Center Development. Immunity.

[12] Hsing L.C., Rudensky A.Y. (2005). The lysosomal cysteine proteases in MHC class II antigen presentation. Immunol. Rev..

[13] Yan X., Wu C., Chen T., Santos M.M., Liu C.-L., Yang C., Zhang L., Ren J., Liao S., Guo H. (2017). Cathepsin S inhibition changes regulatory T-cell activity in regulating bladder cancer and immune cell proliferation and apoptosis. Mol. Immunol..

[14] Prunk M., Kos J. (2018). Nanoparticle Based Delivery of Protease Inhibitors to Cancer Cells. Curr. Med. Chem..

[15] Kos J., Vizin T., Fonović U.P., Pišlar A. (2015). Intracellular signaling by cathepsin X: Molecular mechanisms and diagnostic and therapeutic opportunities in cancer. Semin. Cancer Biol..

[16] Kos J., Lah T.T. (1998). Cysteine proteinases and their endogenous inhibitors: Target proteins for prognosis, diagnosis and therapy in cancer (review). Oncol. Rep..

[17] Mohamed M.M., Sloane B.F. (2006). Cysteine cathepsins: Multifunctional enzymes in cancer. Nat. Rev. Cancer.

[18] Sudhan D., Siemann D.W. (2015). Cathepsin L targeting in cancer treatment. Pharmacol. Ther..

[19] Gocheva V., Wang H.-W., Gadea B.B., Shree T., Hunter K.E., Garfall A.L., Berman T., Joyce J.A. (2010). IL-4 induces cathepsin protease activity in tumor-associated macrophages to promote cancer growth and invasion. Genes Dev..

[20] Kostoulas G., Lang A., Nagase H., Baici A. (1999). Stimulation of angiogenesis through cathepsin B inactivation of the tissue inhibitors of matrix metalloproteinases. FEBS Lett..

[21] Brömme D., Wilson S. (2011). Role of Cysteine Cathepsins in Extracellular Proteolysis. Extracellular Matrix Degradation.

[22] Wang B., Sun J., Kitamoto S., Yang M., Grubb A., Chapman H.A., Kalluri R., Shi G.-P. (2005). Cathepsin S Controls Angiogenesis and Tumor Growth via Matrix-derived Angiogenic Factors. J. Biol. Chem..

[23] Byrne S.M., Aucher A., Alyahya S., Elder M., Olson S.T., Davis D.M., Ashton-Rickardt P.G. (2012). Cathepsin B controls the persistence of memory CD8+ T lymphocytes. J. Immunol..

[24] Gounaris E., Tung C.-H., Restaino C., Maehr R., Köhler R., Joyce J.A., Plough H.L., Barrett T.A., Weissleder R., Khazaie K. (2008). Live Imaging of Cysteine-Cathepsin Activity Reveals Dynamics of Focal Inflammation, Angiogenesis, and Polyp Growth. PLoS ONE.

[25] Herroon M.K., Rajagurubandara E., Rudy D.L., Chalasani A., Hardaway A.L., Podgorski I. (2012). Macrophage cathepsin K promotes prostate tumor progression in bone. Oncogene.

[26] Plüger E.B.E., Boes M., Alfonso C., Schröter C.J., Kalbacher H., Ploegh H.L., Driessen C. (2002). Specific role for cathepsin S in the generation of antigenic peptides in vivo. Eur. J. Immunol..

[27] Wilkinson R., Magorrian S.M., Williams R., Young A., Small D.M., Scott C.J., Burden R.E. (2015). CCL2 is transcriptionally controlled by the lysosomal protease cathepsin S in a CD74-dependent manner. Oncotarget.

[28] Jevnikar Z., Obermajer N., Bogyo M., Kos J. (2008). The role of cathepsin X in the migration and invasiveness of T lymphocytes. J. Cell Sci..

[29] Somoza J.R., Zhan H., Bowman K.K., Yu L., Mortara K.D., Palmer J.T., Clark J.M., McGrath M.E. (2000). Crystal Structure of Human Cathepsin V. Biochemistry.

[30] Kopitar G., Dolinar M., Strukelj B., Pungerčar J., Turk V. (1996). Folding and Activation of Human Procathepsin S from Inclusion Bodies Produced in Escherichia coli. JBIC J. Biol. Inorg. Chem..

[31] Shi G.P., Munger J.S., Meara J.P., Rich D.H., Chapman H.A. (1992). Molecular cloning and expression of human alveolar macrophage cathepsin S, an elastinolytic cysteine protease. J. Biol. Chem..

[32] Pauly T.A., Sulea T., Ammirati M., Sivaraman J., Danley D.E., Griffor M.C., Kamath A.V., Wang I.-K., Laird E.R., Seddon A.P. (2003). Specificity Determinants of Human Cathepsin S Revealed by Crystal Structures of Complexes. Biochemistry.

[33] Kirschke H., Wiederanders B., Brömme D., Rinne A. (1989). Cathepsin S from bovine spleen. Purification, distribution, intracellular localization and action on proteins. Biochem. J..

[34] Shi G.P., Webb A.C., Foster K.E., Knoll J.H., Lemere C.A., Munger J.S., Chapman H.A. (1994). Human cathepsin S: Chromosomal localization, gene structure, and tissue distribution. J. Biol. Chem..

[35] Liu W.-L., Liu D., Cheng K., Liu Y.-J., Xing S., Chi P.-D., Liu X.-H., Xue N., Lai Y.-Z., Guo L. (2016). Evaluating the diagnostic and prognostic value of circulating cathepsin S in gastric cancer. Oncotarget.

[36] Yixuan Y., Kiat L.S., Yee C.L., Huiyin L., Yunhao C., Kuan C.P., Hassan A., Ting W.T., Manuel S.-T., Guan Y.K. (2010). Cathepsin S Mediates Gastric Cancer Cell Migration and Invasion via a Putative Network of Metastasis-Associated Proteins. J. Proteome Res..

[37] Sevenich L., Bowman R.L., Mason S.D., Quail D.F., Rapaport F., Elie B.T., Brogi E., Brastianos P.K., Hahn W.C., Holsinger L.J. (2014). Analysis of tumour- and stroma-supplied proteolytic networks reveals a brain-metastasis-promoting role for cathepsin S. Nat. Cell Biol..

[38] Yang M., Liu J., Shao J., Qin Y., Ji Q., Zhang X., Du J. (2014). Cathepsin S-mediated autophagic flux in tumor-associated macrophages accelerate tumor development by promoting M2 polarization. Mol. Cancer.

[39] Burden R.E., Gormley J.A., Kuehn D., Ward C., Kwok H.F., Gazdoiu M., McClurg A., Jaquin T.J., Johnston J.A., Scott C.J. (2012). Inhibition of Cathepsin S by Fsn0503 enhances the efficacy of chemotherapy in colorectal carcinomas. Biochemistry.

[40] Burden R.E., Gormley J.A., Jaquin T.J., Small D.M., Quinn D.J., Hegarty S.M., Ward C., Walker B., Johnston J.A., Olwill S.A. (2009). Antibody-Mediated Inhibition of Cathepsin S Blocks Colorectal Tumor Invasion and Angiogenesis. Clin. Cancer Res..

[41] Fan Q., Wang X., Zhang H., Li C., Fan J., Xu J. (2012). Silencing cathepsin S gene expression inhibits growth, invasion and angiogenesis of human hepatocellular carcinoma in vitro. Biochem. Biophys. Res. Commun..

[42] Lang Z., Xu J., Li N., Ke Z., Liu R., Maubach G. (2009). Cathepsin S is aberrantly overexpressed in human hepatocellular carcinoma. Mol. Med. Rep..

[43] Ryschich E., Lizdenis P., Ittrich C., Benner A., Stahl S.H., Hamann A., Schmidt J., Knolle P.A., Arnold B., Hämmerling G.J. (2006). Molecular Fingerprinting and Autocrine Growth Regulation of Endothelial Cells in a Murine Model of Hepatocellular Carcinoma. Cancer Res..

[44] Otto H.-H., Schirmeister T. (1997). Cysteine Proteases and Their Inhibitors. Chem. Rev..

[45] Lecaille F., Kaleta J., Brömme D. (2002). Human and Parasitic Papain-Like Cysteine Proteases: Their Role in Physiology and Pathology and Recent Developments in Inhibitor Design. Chem. Rev..

[46] Kang K., Kim W. (2002). Recent developments of cathepsin inhibitors and their selectivity. Expert Opin. Ther. Pat..

[47] Leung-Toung R., Li W., Tam T., Kaarimian K. (2002). Thiol-Dependent Enzymes and Their Inhibitors: A Review. Curr. Med. Chem..

[48] Leroy V. (2004). Cathepsin S inhibitors. Expert Opin. Ther. Pat..

[49] Gauthier J.Y., Black W.C., Courchesne I., Cromlish W., Desmarais S., Houle R., Lamontagne S., Li C.S., Massé F., McKay D.J. (2007). The identification of potent, selective, and bioavailable cathepsin S inhibitors. Bioorganic Med. Chem. Lett..

[50] Lee-Dutra A., Wiener D.K., Sun S. (2011). Cathepsin S inhibitors: 2004–2010. Expert Opin. Ther. Pat..

[51] Sterling T., Irwin J.J. (2015). ZINC 15 – Ligand Discovery for Everyone. J. Chem. Inf. Model..

[52] Wiener J.J.M., Sun S., Thurmond R.L. (2010). Recent advances in the design of cathepsin S inhibitors. Curr. Top. Med. Chem..

[53] Ward Y.D., Thomson D.S., Frye L.L., Cywin C.L., Morwick T., Emmanuel M.J., Zindell R., McNeil D., Bekkali Y., Hrapchak M. (2002). Design and Synthesis of Dipeptide Nitriles as Reversible and Potent Cathepsin S Inhibitors. J. Med. Chem..

[54] Tully D.C., Liu H., Alper P.B., Chatterjee A.K., Epple R., Roberts M.J., Williams J.A., Nguyen K.T., Woodmansee D.H., Tumanut C. (2006). Synthesis and evaluation of arylaminoethyl amides as noncovalent inhibitors of cathepsin S. Part 3: Heterocyclic P3. Bioorganic Med. Chem. Lett..

[55] Cai J., Fradera X., Van Zeeland M., Dempster M., Cameron K.S., Bennett D.J., Robinson J., Popplestone L., Baugh M., Westwood P. (2010). 4-(3-Trifluoromethylphenyl)-pyrimidine-2-carbonitrile as cathepsin S inhibitors: N3, not N1 is critically important. Bioorganic Med. Chem. Lett..

[56] Jadhav P.K., Schiffler M.A., Gavardinas K., Kim E.J., Matthews D.P., Staszak M.A., Coffey D.S., Shaw B.W., Cassidy K.C., Brier R.A. (2014). Discovery of Cathepsin S Inhibitor LY3000328 for the Treatment of Abdominal Aortic Aneurysm. ACS Med. Chem. Lett..

[57] Hilpert H., Mauser H., Humm R., Anselm L., Kuehne H., Hartmann G., Gruener S., Banner D.W., Benz J., Gsell B. (2013). Identification of Potent and Selective Cathepsin S Inhibitors Containing Different Central Cyclic Scaffolds. J. Med. Chem..

[58] Ahmad S., Bhagwati S., Kumar S., Banerjee D., Siddiqi M.I. (2020). Molecular modeling assisted identification and biological evaluation of potent cathepsin S inhibitors. J. Mol. Graph. Model..

[59] Kerns J.K., Nie H., Bondinell W., Widdowson K.L., Yamashita D.S., Rahman A., Podolin P.L., Carpenter D.C., Jin Q., Riflade B. (2011). Azepanone-based inhibitors of human cathepsin S: Optimization of selectivity via the P2 substituent. Bioorganic Med. Chem. Lett..

[60] Kim M., Jeon J., Song J., Suh K.H., Kim Y.H., Min K.-H., Lee K.-O. (2013). Synthesis of proline analogues as potent and selective cathepsin S inhibitors. Bioorganic Med. Chem. Lett..

[61] Wilkinson R., Young A., Burden R.E., Williams R., Scott C.J. (2016). A bioavailable cathepsin S nitrile inhibitor abrogates tumor development. Mol. Cancer.

[62] Tber Z., Wartenberg M., Jacques J.-E., Roy V., Lecaille F., Warszycki D., Bojarski A.J., Lalmanach G., Agrofoglio L.A. (2018). Selective inhibition of human cathepsin S by 2,4,6-trisubstituted 1,3,5-triazine analogs. Bioorganic Med. Chem..

[63] Farokhzad O.C., Langer R. (2009). Impact of Nanotechnology on Drug Delivery. Acs Nano.

[64] Peer D., Karp J.M., Hong S., Farokhzad O.C., Margalit R., Langer R. (2007). Nanocarriers as an emerging platform for cancer therapy. Nat. Nanotechnol..

[65] Wang A.Z., Langer R., Farokhzad O.C. (2012). Nanoparticle Delivery of Cancer Drugs. Annu. Rev. Med..

[66] Matsumura Y., Maeda H. (1986). A new concept for macromolecular therapeutics in cancer chemotherapy: Mechanism of tumoritropic accumulation of proteins and the antitumor agent smancs. Cancer Res..

[67] Maeda H. (2015). Toward a full understanding of the EPR effect in primary and metastatic tumors as well as issues related to its heterogeneity. Adv. Drug Deliv. Rev..

[68] Fang J., Islam W., Maeda H. (2020). Exploiting the dynamics of the EPR effect and strategies to improve the therapeutic effects of nanomedicines by using EPR effect enhancers. Adv. Drug Deliv. Rev..

[69] Toth I., Skwarczynski M. (2014). The immune system likes nanotechnology. Nanomedicine.

[70] Lepeltier E., Nuhn L., Lehr C., Zentel R. (2015). Not just for tumor targeting: Unmet medical needs and opportunities for nanomedicine. Nanomedicine.

[71] Irvine D.J., Dane E.L. (2020). Enhancing cancer immunotherapy with nanomedicine. Nat. Rev. Immunol..

[72] Martin J.D., Cabral H., Stylianopoulos T., Jain R.K. (2020). Improving cancer immunotherapy using nanomedicines: Progress, opportunities and challenges. Nat. Rev. Clin. Oncol..

[73] Lybaert L., Vermaelen K., de Geest B.G., Nuhn L. (2018). Immunoengineering through cancer vaccines—A personalized and multi-step vaccine approach towards precise cancer immunity. J. Control. Release.

[74] Duncan R., Gaspar R.S. (2011). Nanomedicine(s) under the Microscope. Mol. Pharm..

[75] Anselmo A.C., Mitragotri S. (2019). Nanoparticles in the clinic: An update. Bioeng. Transl. Med..

[76] Milla P., Dosio F., Cattel L. (2012). PEGylation of proteins and liposomes: A powerful and flexible strategy to improve the drug delivery. Curr. Drug Metab..

[77] Knop K., Hoogenboom R., Fischer D., Schubert U.S. (2010). Poly(ethylene glycol) in Drug Delivery: Pros and Cons as Well as Potential Alternatives. Angew. Chem. Int. Ed..

[78] Cabral H., Miyata K., Osada K., Kataoka K. (2018). Block Copolymer Micelles in Nanomedicine Applications. Chem. Rev..

[79] Kim T.-Y. (2004). Phase I and Pharmacokinetic Study of Genexol-PM, a Cremophor-Free, Polymeric Micelle-Formulated Paclitaxel, in Patients with Advanced Malignancies. Clin. Cancer Res..

[80] Sah H., Thoma L.A., Desu H.R., Sah E., Wood G.C. (2013). Concepts and practices used to develop functional PLGA-based nanoparticulate systems. Int. J. Nanomed..

[81] Cogo F., Williams R., Burden R.E., Scott C.J. (2019). Application of nanotechnology to target and exploit tumour associated proteases. Biochemistry.

[82] Cegnar M., Kos J., Kristl J. (2004). Cystatin incorporated in poly(lactide-co-glycolide) nanoparticles: Development and fundamental studies on preservation of its activity. Eur. J. Pharm. Sci..

[83] Cegnar M., Premzl A., Zavašnik-Bergant V., Kristl J., Kos J. (2004). Poly(lactide-co-glycolide) nanoparticles as a carrier system for delivering cysteine protease inhibitor cystatin into tumor cells. Exp. Cell Res..

[84] Cegnar M., Kos J., Kristl J. (2006). Intracellular delivery of cysteine protease inhibitor cystatin by polymeric nanoparticles. J. Nanosci. Nanotechnol..

[85] Varshosaz J., Fard M.M., Mirian M., Hassanzadeh F. (2020). Targeted Nanoparticles for Co-delivery of 5-FU and Nitroxoline, a Cathepsin B Inhibitor, in HepG2 Cells of Hepatocellular Carcinoma. Anti-Cancer Agents Med. Chem..

[86] Yu N.Y., Fathi A., Murphy C.M., Mikulec K., Peacock L., Cantrill L.C., Dehghani F., Little D.G., Schindeler A. (2015). Local co-delivery of rh BMP -2 and cathepsin K inhibitor L006235 in poly(d,l -lactide- co -glycolide) nanospheres. J. Biomed. Mater. Res. Part B Appl. Biomater..

[87] Kumar V., Wang L., Riebe M., Tung H.-H., Prud’Homme R.K. (2009). Formulation and Stability of Itraconazole and Odanacatib Nanoparticles: Governing Physical Parameters. Mol. Pharm..

[88] Mikhaylov G., Mikac U., Magaeva A.A., Itin V.I., Naiden E.P., Psakhye I., Babes L., Reinheckel T., Peters C., Zeiser R. (2011). Ferri-liposomes as an MRI-visible drug-delivery system for targeting tumours and their microenvironment. Nat. Nanotechnol..

[89] Mikhaylov G., Klimpel D., Schaschke N., Mikac U., Vizovišek M., Fonović M., Turk V., Turk B., Vasiljeva O. (2014). Selective Targeting of Tumor and Stromal Cells By a Nanocarrier System Displaying Lipidated Cathepsin B Inhibitor**. Angew. Chem. Int. Ed..

[90] Bratovš A., Kramer L., Mikhaylov G., Vasiljeva O., Turk B. (2019). Stefin A-functionalized liposomes as a system for cathepsins S and L-targeted drug delivery. Biochemistry.

[91] Tabish T.A., Pranjol Z.I., Whatmore J.L., Zhang S. (2020). Status and Future Directions of Anti-metastatic Cancer Nanomedicines for the Inhibition of Cathepsin L. Front. Nanotechnol..

[92] Junior J.C.Q., Carlos F.D.R.R., Montanari A., Leitão A., Mignone V.W., Arruda M.A., Turyanska L., Bradshaw T.D. (2019). Apoferritin encapsulation of cysteine protease inhibitors for cathepsin L inhibition in cancer cells. RSC Adv..

[93] Wang N., Pechar M., Li W., Kopečková P., Brömme D., Kopeček J. (2002). Inhibition of Cathepsin K with Lysosomotropic Macromolecular Inhibitors. Biochemistry.

[94] Wang D., Li W., Pechar M., Kopečková P., Brömme D., Kopeček J. (2004). Cathepsin K inhibitor–polymer conjugates: Potential drugs for the treatment of osteoporosis and rheumatoid arthritis. Int. J. Pharm..

[95] Hemmelmann M., Mohr K., Fischer K., Zentel R., Schmidt M. (2013). Interaction of pHPMA–pLMA Copolymers with Human Blood Serum and Its Components. Mol. Pharm..

[96] Alberg I., Kramer S., Schinnerer M., Hu Q., Seidl C., Leps C., Drude N., Möckel D., Rijcken C., Lammers T. (2020). Polymeric Nanoparticles: Polymeric Nanoparticles with Neglectable Protein Corona (Small 18/2020). Small.

[97] Talelli M., Barz M., Rijcken C.J., Kiessling F., Hennink W.E., Lammers T. (2015). Core-crosslinked polymeric micelles: Principles, preparation, biomedical applications and clinical translation. Nano Today.

[98] Leber N., Nuhn L., Zentel R. (2017). Cationic Nanohydrogel Particles for Therapeutic Oligonucleotide Delivery. Macromol. Biosci..

[99] Stickdorn J., Nuhn L. (2020). Reactive-ester derived polymer nanogels for cancer immunotherapy. Eur. Polym. J..

[100] Nuhn L., Braun L., Overhoff I., Kelsch A., Schaeffel D., Koynov K., Zentel R. (2014). Degradable Cationic Nanohydrogel Particles for Stimuli-Responsive Release of siRNA. Macromol. Rapid Commun..

[101] Nuhn L., Van Herck S., Best A., Deswarte K., Kokkinopoulou M., Lieberwirth I., Koynov K., Lambrecht B.N., De Geest B.G. (2018). FRET Monitoring of Intracellular Ketal Hydrolysis in Synthetic Nanoparticles. Angew. Chem. Int. Ed..

[102] Nuhn L., Vanparijs N., De Beuckelaer A., Lybaert L., Verstraete G., Deswarte K., Lienenklaus S., Shukla N.M., Salyer A.C.D., Lambrecht B.N. (2016). pH-degradable imidazoquinoline-ligated nanogels for lymph node-focused immune activation. Proc. Natl. Acad. Sci. USA.

[103] Nuhn L., Van Hoecke L., Deswarte K., Schepens B., Li Y., Lambrecht B.N., De Koker S., David S.A., Saelens X., De Geest B.G. (2018). Potent anti-viral vaccine adjuvant based on pH-degradable nanogels with covalently linked small molecule imidazoquinoline TLR7/8 agonist. Biomaterials.

[104] Nuhn L., De Koker S., Van Lint S., Zhong Z., Catani J.P., Combes F., Deswarte K., Li Y., Lambrecht B.N., Lienenklaus S. (2018). Nanoparticle-Conjugate TLR7/8 Agonist Localized Immunotherapy Provokes Safe Antitumoral Responses. Adv. Mater..

[105] Nuhn L., Bolli E., Massa S., Vandenberghe I., Movahedi K., Devreese B., Van Ginderachter J.A., De Geest B.G., Boli E. (2018). Targeting Protumoral Tumor-Associated Macrophages with Nanobody-Functionalized Nanogels through Strain Promoted Azide Alkyne Cycloaddition Ligation. Bioconjugate Chem..

[106] Obermajer N., Kocbek P., Repnik U., KuŽNik A., Cegnar M., Kristl J., Kos J. (2007). Immunonanoparticles—An effective tool to impair harmful proteolysis in invasive breast tumor cells. FEBS J..

[107] Kos J., Obermajer N., Doljak B., Kocbek P., Kristl J. (2009). Inactivation of harmful tumour-associated proteolysis by nanoparticulate system. Int. J. Pharm..

[108] Tuettenberg A., Steinbrink K., Schuppan D. (2016). Myeloid cells as orchestrators of the tumor microenvironment: Novel targets for nanoparticular cancer therapy. Nanomedicne.

[109] Schupp J., Krebs F.K., Zimmer N., Trzeciak E., Schuppan D., Tuettenberg A. (2019). Targeting myeloid cells in the tumor sustaining microenvironment. Cell. Immunol..

[110] Leber N., Kaps L., Yang A., Aslam M., Giardino M., Klefenz A., Choteschovsky N., Rosigkeit S., Mostafa A., Nuhn L. (2019). α-Mannosyl-Functionalized Cationic Nanohydrogel Particles for Targeted Gene Knockdown in Immunosuppressive Macrophages. Macromol. Biosci..

[111] Scodeller P., Simón-Gracia L., Kopanchuk S., Tobi A., Kilk K., Säälik P., Kurm K., Squadrito M.L., Kotamraju V.R., Rinken A. (2017). Precision Targeting of Tumor Macrophages with a CD206 Binding Peptide. Sci. Rep..

[112] Lepland A., Asciutto E.K., Malfanti A., Simón-Gracia L., Sidorenko V., Vicent M.J., Teesalu T., Scodeller P. (2020). Targeting Pro-Tumoral Macrophages in Early Primary and Metastatic Breast Tumors with the CD206-Binding mUNO Peptide. Mol. Pharm..

[113] Wagener K., Bros M., Krumb M., Langhanki J., Pektor S., Worm M., Schinnerer M., Montermann E., Miederer M., Frey H. (2020). Targeting of Immune Cells with Trimannosylated Liposomes. Adv. Ther..

[114] Wong C.S., Hoogendoorn S., Van Der Marel G.A., Overkleeft H.S., Codée J.D.C. (2015). Targeted Delivery of Fluorescent High-Mannose-Type Oligosaccharide Cathepsin Inhibitor Conjugates. ChemPlusChem.

